# Epigenetics and Inflammation in Diabetic Nephropathy

**DOI:** 10.3389/fphys.2021.649587

**Published:** 2021-05-05

**Authors:** Bao-Yi Shao, Shao-Fei Zhang, Hai-Di Li, Xiao-Ming Meng, Hai-Yong Chen

**Affiliations:** ^1^Li Ka Shing Faculty of Medicine, The University of Hong Kong, Hong Kong, China; ^2^Inflammation and Immune Mediated Diseases Laboratory of Anhui Province, Anhui Institute of Innovative Drugs, School of Pharmacy, Anhui Medical University, Hefei, China; ^3^School of Chinese Medicine, The University of Hong Kong, Hong Kong, China; ^4^Department of Chinese Medicine, The University of Hong Kong-Shenzhen Hospital, Shenzhen, China

**Keywords:** diabetic nephropathy, epigenetics, DNA methylation, histone modifications, non-coding RNAs, inflammation

## Abstract

Diabetic nephropathy (DN) leads to high morbidity and disability. Inflammation plays a critical role in the pathogenesis of DN, which involves renal cells and immune cells, the microenvironment, as well as extrinsic factors, such as hyperglycemia, chemokines, cytokines, and growth factors. Epigenetic modifications usually regulate gene expression via DNA methylation, histone modification, and non-coding RNAs without altering the DNA sequence. During the past years, numerous studies have been published to reveal the mechanisms of epigenetic modifications that regulate inflammation in DN. This review aimed to summarize the latest evidence on the interplay of epigenetics and inflammation in DN, and highlight the potential targets for treatment and diagnosis of DN.

## Introduction

The latest Diabetes Atlas by the International Diabetes Federation indicates that the current number of patients with diabetes mellitus (DM) is 463 million in 2019, which is estimated to increase to 578 million by 2030 and to 700 million by 2045 (International Diabetes Federation, 2019). DM and its complications seriously affect patients’ quality of life and result in tremendous socioeconomic burdens (GBD, 2017 Disease and injury incidence and prevalence collaborators, 2018; Lin et al., 2020b). Diabetic nephropathy (DN), one of the most common microvascular complications of DM, is the major contributor to chronic kidney disease (CKD) and end-stage renal disease (Ruiz-Ortega et al., 2020). Approximately 30–40% of DM patients gradually develop DN (Lim, 2014). Current therapies, including intensive glucose control and the treatment of hypertension through renin-angiotensin-aldosterone system (RAAS) blockers, only slow down the progression of DN and fail to reverse or stop it (Sanz et al., 2019; Ruiz-Ortega et al., 2020). Therefore, early diagnosis and novel treatment for DN are of great significance while recognizing its etiology remains urgent.

The biologist Conrad Waddington firstly introduced ‘epigenetics’ which describes a phenomenon of inheritance that is independent of DNA sequence (Russo et al., 1996; Goldberg et al., 2007). This concept has become one of the frontiers of genetic research over the years. Epigenetic modifications modulate gene expressions through DNA methylation, histone modification, and non-coding RNAs involving in the pathogenesis of DN (Keating and El-Osta, 2013; Reddy et al., 2015). Studies have also shown that the modifications are reversible indicating potential therapeutic value for DN (Hotamisligil, 2017; Kato and Natarajan, 2019). Low grade chronic inflammation is a major characteristic in the pathogenesis of DN, but the pathophysiological relevance between epigenetics and inflammation has not been fully summarized. In this review, we highlighted recent epigenetic modifications relevant to inflammation and its signaling pathways in DN. The prespecified search strategies were shown in the Supplementary Material 1.

## Inflammation in the Progression of DN

### The Role of Renal Resident and Immune Cells in the Inflammatory Response

**FIGURE 1:**
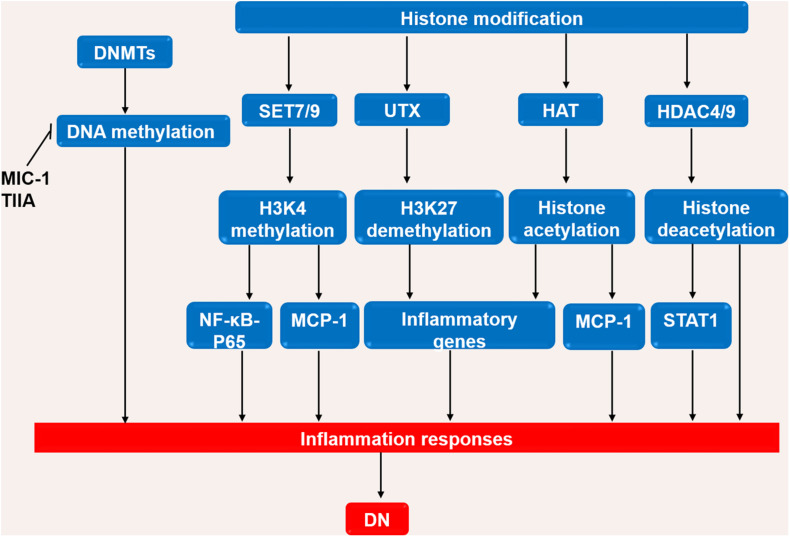
Interaction of immune cells, kidney intrinsic cells and epigenetic modifications. DNA methylation, histone modifications, and non-coding RNA modifications activate inflammatory pathways by interactions of immune cells and kidney intrinsic cells. OS, oxidative stress; ROS, reactive oxygen species; HG, high glucose; Ang II, angiotensin II; AGE-RAGE, advanced glycation end-products-receptor for advanced glycation end products; IL, interleukin; TNF, tumor necrosis factor; MCP-1, monocyte chemoattractant protein 1; NF-κB, nuclear factor-κB; JAK-STAT, Janus kinase/signal transducer and activator of transcription; NRF2, Nuclear Factor-2 Erythroid Related Factor; NLRP, NOD-like receptor pyrin domain-containing protein.

Hyperglycemia and glucose metabolites such as advanced glycation end products (AGEs) have long been regarded as initial factors of DN which promote the loss of podocytes, the hyperfiltration of endothelial cells, the expansion of mesangial cells and the thickening of glomerular basement membrane, and finally result in the deposition of extracellular matrix in the glomerulus (Schena and Gesualdo, 2005; Grabias and Konstantopoulos, 2014). The injured resident cells in kidney release chemokines and cytokines to attract the infiltration of immune cells (e.g., monocytes, macrophages, dendritic cells, and lymphocytes) (Tang and Yiu, 2020). Macrophages/monocytes are found to be the most predominant immune cells through both clinical and experimental studies. Previous study shows that macrophages are positively associated with pathological lesions in DN (Chow et al., 2004). A recent study of single cell RNA sequencing (scRNA-seq) indicates proportions of endothelial cells and immune cells are significantly increased while mesangial cells and podocytes are decreased in the glomerular cells in diabetic mouse kidney (Fu et al., 2019a). Among of immune cells in this study, macrophages are predominant, particularly M1 phenotype macrophages (Fu et al., 2019a). It has been also demonstrated that infiltration of macrophages in the glomeruli and tubulointerstitial tissues was increased in renal biopsies of patients (Klessens et al., 2017). In addition, the depletion of macrophages significantly reduces proteinuria and glomerular pathological changes in diabetic mice (You et al., 2013). The scRNA-seq analysis of kidney cortex from diabetic (*n* = 3) and non-diabetic patients (*n* = 3) shows patients in early diabetic nephropathy have 78 folds of leukocytes, including T cells, B cells, monocytes and plasma cells, compared to non-diabetic patients (Wilson et al., 2019). Few macrophages are observed in early diabetic kidneys (Wilson et al., 2019). Proportions of kidney cells and immune cells, and their roles at different stages of DN needed to be further studies. Epigenetic modifications in diabetic kidneys are shown in Figure 1.

The infiltration of macrophages is promoted by chemokines and adhesion molecules which are released from resident cells under the stimulation of high glucose and AGEs (Hickey and Martin, 2013). Notably, MCP-1 is an important mediator in the infiltration of macrophages and the progression of inflammation (Chow et al., 2006). The deletion of MCP-1 in mice and inhibition of MCP-1 in type 2 diabetic patients have been shown to improve renal function (Chow et al., 2006). Previous studies have shown that an increase in M1 macrophages is negatively associated with renal function (Wang et al., 2017), while the induction of M2 macrophages has been shown to attenuate renal damage in DN mouse model (Sun et al., 2015). High glucose and AGEs promote macrophages to M1 polarization and the release of inflammatory cytokines, such as tumor necrosis factor (TNF), contributing to pathogenesis in the early stage of diabetes (Webster et al., 1997). Additionally, macrophages can also act as myofibroblasts through the process of macrophage-myofibroblast transition (MMT) to deteriorate renal fibrosis, replace parenchyma tissue with (Tang et al., 2020b) extracellular matrix (ECM), and also contribute to the production of reactive oxygen species (ROS) and proteases (Meng et al., 2014; Torres et al., 2020). Orchestrated by TGF-β/Smad signaling pathway, MMT is a newly known fibrosis process which has been rarely found neither in acute inflammation, nor in normal kidney, indicating that chronic inflammation was the principle contributor to fibrosis (Meng et al., 2016; Tang et al., 2019). A recent study has found that brain-specific transcription factor POU4F1 is the only transcription factor taking part in the TGF-β1/Smad3-driven MMT and thus could be a new therapeutic target in chronic inflammation induced MMT fibrosis (Tang et al., 2020b). The proto-oncogene tyrosine protein kinase *SRC* presents as a direct SMAD3 target gene and is also essential for MMT in macrophages (Tang et al., 2018b). In general, the accumulation of macrophage are not only related to the degree of inflammation and kidney function, but also correlated to glomerulosclersis and the degree of interstitial fibrosis (Tang et al., 2019). Studies have shown that aberrant intrarenal infiltration and activation of T cells are involved in the pathogenesis of DN in both clinical samples and streptozotocin (STZ)-induced diabetes mice (Moon et al., 2012). Clinical findings show that T cell immunity and TNF-α signaling pathway are activated during the early development of DN in patients (Moon et al., 2012; Lampropoulou et al., 2020). The proportions of T helper cells (Th1, Th2, Th17 and regulatory T (Treg) cells) in DN are altered with the increased levels of Th1 and Th17, and the decreased level of Treg (Zhang et al., 2014). Adoptive transfer of CD4 + Foxp3 + Treg cells in mice have been found to ameliorate diabetic kidney injuries and insulin resistance by inhibiting inflammation (Eller et al., 2011).

### The Role of Inflammatory Mediators and Signaling Pathways in DN

**FIGURE 2:**
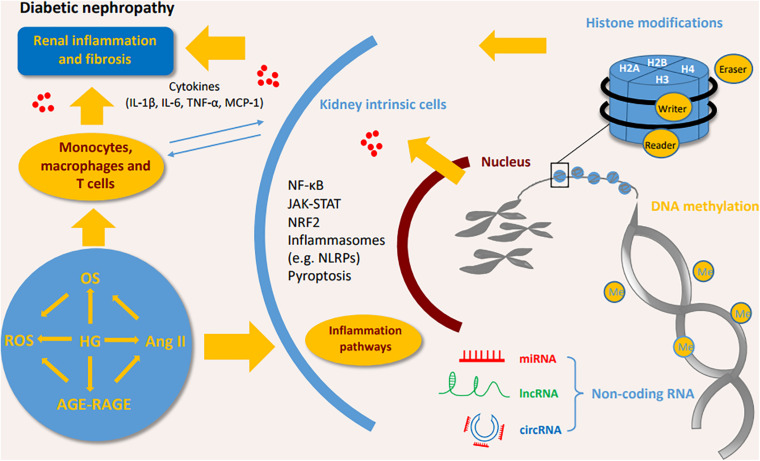
The inflammatory pathways involved in the DN process. High glucose or stimulating factors cause the activation of multiple pathways, including P38/MAPK, PI3K/AKT, TLR4/NLRP3 and the novel CRP/DPP4/CD32b, which all further activate NF-κB pathway, and transcriptionally promote the expression of multiple inflammatory cytokines to enhance inflammation. SMAD7 can inhibit NF-κB activity. NRF2 has anti-inflammatory and antioxidant functions in DN. In addition, JAK/STAT pathway induces IL-6 to promote the inflammatory response in DN. DN, diabetic nephropathy; IL, interleukin; TNF, tumor necrosis factor; NF-κB, Nuclear factor-κB; MCP-1, monocyte chemoattractant protein-1; STAT, Signal transducer and activator of transcription; NRF2, Nuclear factor-2 erythroid related factor; NLRP3, NOD-like receptor protein 3; ROS, reactive oxygen species; DPP4, dipeptidyl peptidase-4; MAPK, mitogen-activated protein kinase; JAK2, Janus kinase; TLR4, Toll-like receptors 4; CRP, C-reactive protein; AGE, advanced glycation end product; RAGE, Receptor for advanced glycosylation end products; Ang II, angiotensin II; TGF, Transforming growth factor.

Several signaling pathways contribute to the inflammation and the release of inflammatory cytokines (Figure 2; Newton and Dixit, 2012). Interleukins (ILs) play critical roles in the regulation of the immune system. Studies have shown that the circulating level of IL-6 is positively correlated with the progression of DN in patients (Saraheimo et al., 2003), and IL-1β, IL-18, and IL-17A are associated with the occurrence and development of DN (Cortvrindt et al., 2017; Lemos et al., 2018; Lin et al., 2020a). TNF-α is involved in the development of various diseases, such as psoriasis, rheumatoid arthritis, and CKD (Elliott et al., 1994; Pina et al., 2016). Studies have demonstrated that macrophages are the main source of renal TNF-α (Awad et al., 2015). In diabetic mice, the inhibition of TNF-α leads to decreased urinary albumin excretion, and in a clinical trial where DN patients were treated with pentoxifylline, a methylxanthine derivative with anti-inflammatory function, the reduction in urinary TNF-α concentration was directly correlated with the change in albuminuria, suggesting the role of TNF-α in the pathogenesis of DN (Moriwaki et al., 2007; Navarro-González et al., 2015).

Nuclear factor-κB (NF-κB) is the basic transcription factor that plays a pivotal role in inflammation in DN patients. Activated by upstream signals such as AGEs, angiotensin II, and oxidative stress (OS), NF-κB dissociates from its inhibitor IκB proteins and is transferred into the nucleus to regulate the expression of inflammatory gene including cytokines, chemokines, and adhesion molecules such as IL-6, TNF-α, and MCP-1 (Wada and Makino, 2016; Mikuda et al., 2018). One of the upstream signal pathways stimulated by AGEs is called the p38 mitogen-activated protein kinase (MAPK) pathway (Wu et al., 2002). The p38 MAPK pathway induces the activation of NF-κB in the infiltrating macrophages of DN (Adhikary et al., 2004). In turn, in renal parenchymal cells, elevated IL-1 and TNF-α have been shown to promote the phosphorylation of p38 MAPK, demonstrating their inflammatory roles in DN (Adhikary et al., 2004). Similarly, PI3K/AKT/mTOR is a widely studied signaling pathway that mediates the phenotype and injury of podocytes in DN. Stimulated by AGEs, PI3K/AKT can also promote NF-κB and aggravate inflammation (Ahmad et al., 2013; Hong et al., 2017). Recently, C-reactive protein (CRP) has been found to trigger a novel NF-κB-involved signaling pathway in the progression of DN, more narrowly, in human CRP transfected-db/db mice and cultured renal tubular epithelial cells, CRP is proved to promote inflammation via the evoking and dimerization of dipeptidyl peptidase-4 (DPP4) through DPP4/CD32b/NF-kB signaling circuit. The blockage of the circult by the DPP4 inhibitor, linagliptin, attenuates DN, suggesting the potential therapeutic effect for DN (Tang et al., 2021).

TGF-β/SMAD signaling pathway plays a criticial role in diabetic kidney injuries (Chen et al., 2011, 2014a; Liu et al., 2011; Lan, 2012; Zhong et al., 2013; Li et al., 2014; Zhang et al., 2019b; Xu et al., 2020a; Yang et al., 2020). In the diabetic kidney, high glucose and AGEs enhance the phosphorylation of SMAD3 and decrease the phosphorylation SMAD7. SMAD3 deficiency prevents renal inflammation and fibrosis in SMAD3-db/db mice via regulations of lncRNA Erbb4-IR.transcription and miR-29b (Xu et al., 2020a). SMAD3 deficiency protects against diabetes-associated beta cell dysfunction and loss in DN mice (Sheng et al., 2021). SMAD3 also promotes autophagy dysregulation and kidney injury (Yang et al., 2020). SMAD7 inhibits IκBα, an NF-κB inhibitor, suppressing the activation of NF-κB pathway (Chung et al., 2009). The deletion of SMAD7 significantly aggravates renal inflammation as evidenced by the upregulation of IL-1β, TNF-α, and MCP-1 in diabetic mice by crosstalk with NF-κB pathway, and the addition of SMAD7 attenuates the kidney injuries (Chen et al., 2011). Thus, TGF-β/SMAD and NF-κB crosstalk pathway may act as a novel prevention and therapeutic targets for diabetic nephropathy.

Activation of OS signaling pathways contributes to renal inflammation in DN. Nuclear factor-2 erythroid related factor (NRF2) is a protein that has the ability to alleviate inflammation and act as an antioxidant mediator in the process of OS. NRF2 reduces the infiltration of macrophages and proinflammatory cytokines by ameliorating oxidative overload. Clinical studies have demonstrated that the NRF2 activator, such as bardoxolone methyl, improves kidney function in diabetic patients (Pergola et al., 2011).

The nucleotide-binding oligomerization domain (NOD) family and NOD-like receptor pyrin domain-containing protein (NLRP) family are involved in DN. NOD2 promotes the endothelial-to-mesenchymal transition in DN (Shang et al., 2017). NLRP3 promotes the generation of IL-1β and IL-18 by activating the NLRP3-Caspase-1-IL-1β pathway in diabetic kidneys (Chi et al., 2020; Wang et al., 2020). NLRP3 has interactions with Toll-like receptors, ROS and NF-κB pathway to promote inflammation in DN (Shahzad et al., 2015; Wang et al., 2020).

The Janus kinase/signal transducer and activator of transcription (JAK-STAT) pathway is involved in processing extracellular signals (cytokines and chemokines) to the cell nucleus, resulting in gene expression (O’shea et al., 2013). The clinical findings show that JAK2 is increased in the podocytes of patients with early DN (Berthier et al., 2009). Podocyte-specific JAK2 overexpression in diabetic mice aggravates glomerulopathy while the inhibition of JAK1/2 attenuates the phenotypic changes of diabetic kidney (Zhang et al., 2017).

## DN and Epigenetic Modifications Involved in Inflammation

### DNA Methylation Involved in Inflammation of DN

DNA methylation promotes inflammatory activation of immune cells in diabetic kidney disease and demethylating agents prevent the progressive kidney disease (Larkin et al., 2018; Chen et al., 2019b). DNA methylation is a process in which the methyl group of S-adenosylmethionine is transferred to the cytosine of DNA under the catalysis of DNA methyltransferases (DNMTs), resulting in down-regulation of the gene expression (Yagi et al., 2012). DNMTs mainly include DNMT1, DNMT3a, and DNMT3b. DNMT1 has been found to contribute to the maintenance of methylation and the other DNMTs are related to *de novo* methylation (Hsieh, 1999). DNA methylation occurs specifically at the 5’ site of the CpG dinucleotide cytosine residue, hindering the binding of transcription factors and promoters, subsequently inhibiting transcription (Yagi et al., 2012). The genome-wide DNA methylation analysis shows that DNA methylation is associated the kidney injuries and kidney inflammation in DN patients (VanderJagt et al., 2015; Park et al., 2019). *In vivo* study also indicates high-glucose induced high levels of methylation in kidney cells. It is found that there are 173 differentially methylated regions (DMRs) in high glucose (HG)-treated mesangial cells compared to the low-glucose (LG) treatment (Li et al., 2020d). Suppression of methylation by bioactive constituent extracted from plants, e.g., moringa isothiocyanate (MIC-1), potentially down-regulates expression of *TGF-*β*1*, and changes the *Nrf2, Col4a2, Tceal3, Ret, and Agt* expressions (Li et al., 2020d; Cheng et al., 2019).

Aberrant cytosine methylation of the upstream regulators of the mammalian target of rapamycin (mTOR) promotes inflammation by the upregulation of DNMT1 in DN (Chen et al., 2019b). Notably, DNA methylation is dynamic and can be altered by environmental factors. Studies have found that hyperglycemia in T2DM patients triggers a self-regulatory mechanism leading to the reduction of 5mC levels in the peripheral blood, which indicates that the DNA might undergo demethylation via the upregulation of ten-eleven-translocation 2 (TET2), a DNA demethylation enzyme (Yuan et al., 2019).

### Histone Modifications Involved in Inflammation of DN

The nucleosome is the basic unit of chromatin consisting of DNA and wrapped histone proteins. The post-translational modifications (PTMs) on chromatin histone include acetylation, ubiquitination, phosphorylation, and methylation. Recently, the genome-wide analysis of chromatin binding proteins and histone modifications has been conducted through chromatin immunoprecipitation followed by high-throughput sequencing (ChIP-seq) or by microarrays. The modifications are mainly mediated by three types of enzymes: writer, eraser, and reader. Writers/erasers carry on the modifications by adding/removing methyl or acetyl groups at amino acid residues in histone, such as histone acetyltransferase, histone methyltransferase (HMT), histone deacetylase (HDAC) and histone demethylase (HDM) (Bhatt et al.). Readers are the effectors that can identify and interpret post-translational modifications. Histone acetylation promotes gene transcription, while histone methylation promotes or inhibits gene transcription (Kouzarides, 2007). Specifically, the methylation of histone mostly happens on the residues of lysine and arginine. There are three types of methylation in lysine, namely monomethylation, dimethylation and trimethylation, and all three types of methylation of H3 at lysine 4 (H3K4me1, H3K4me2 and H3K4me3, respectively) exert an active effect (Kato and Natarajan, 2019). Similarly, H3K36me2 and H3K36me3 are enriched at transcriptional activation genome regions (Kato and Natarajan, 2019). Conversely, the methylation of H3K9me3, H3K27me3 and H4K20me3 are associated with gene repression (Kato and Natarajan, 2019). These modifications usually happen at promoters, insulators, enhancers, and other cis-regulatory regions, and finally lead to aberrant gene expression (Barski et al., 2007; Heintzman et al., 2009; Pradeepa et al., 2016).

Histone PTMs are involved in the pathogenesis of DN (Kato and Natarajan, 2019). HG and other danger signals increase the expression of pro-inflammatory genes by histone PTMs (Kato and Natarajan, 2019). *TXNIP*, pro-inflammatory gene, has been demonstrated to play an important role in the development of DN (Chen et al., 2008). In hyperglycemia-induced DN mice, HG-induced *Txnip* expression is associated with the enrichment of activated histone marks H3K9ac, H3K4me3, H3K4me1, and the repressive histone mark H3K27me3 at the promoter region of the gene, which has also been proved in human mesanginal cells (De Marinis et al., 2016). Furthermore, histone methylation take part in the process of inflammation via the secretion of inflammatory cytokines in diabetes. Specifically, H3K4 methylation could be mediated by HMT SET7 (Cheng et al., 2005). It is reported that transient HG causes the recruitment of HMT SET7 and increases H3K4 methylation at the NF-κB -P65 promoter, which promotes the expression of P65, MCP-1 and VCAM-1 in endothelial cells (El-Osta et al., 2008). Meanwhile, in endoplasmic reticulum (ER) stress induced kidney model of db/db mice, the increased expression of *Mcp-1* is associated with the enrichment of H3K4me1 at *Mcp-1* promoters, and could be significantly attenuated by the methyltransferase SET7/9 gene silencing (Chen et al., 2014c). The other study indicates that SET7/9 modifies chromatin histone lysine at promoters of *MCP-1*and *TNF-*α which promotes the inflammation in THP-1 monocytes (Li et al., 2008). In contrast, UTX (ubiquitously transcribed tetratricopeptide repeat, X chromosome) is a histone demethylase that can remove di- and tri-methyl groups from H3K27 (Choi et al., 2015). Studies have reported that the expression of UTX is upregulated in podocytes, tubular and mesangial cells of DN patients *in vitro* and *in vivo* (Majumder et al., 2018). Moreover, the knockout of UTX or the treatment of UTX inhibitor, GSK-J4, can reduce palmitic acid-induced increase of inflammation and DNA damage (Chen et al., 2019c). Furthermore, one study demonstrated that the inhibition of UTX could inhibit hypertrophy, a key event in glomerular dysfunction (Jia et al., 2019). In parallel, TGF-β down-regulates Enhancer of Zeste homolog 2 (EZH2), a H3K27me3 methyltransferase, by inducing miR-101b, which targets the 3’-untranslated region (3’-UTR) of *EZH2*. Meanwhile, TGF-β up-regulates UTX, a key role for H3K27me3 demethylases in renal mesangial cells. TGF-β-induced the inhibition of H3K27me3 augments pathological genes via dysregulation of associated histone-modifying enzymes and miR-101b in DN (Jia et al., 2019). Another H3K27me3 demethylase JMJD3 regulates inflammatory genes in macrophages (De Santa et al., 2007). To conclude, these studies suggest that the inhibition of H3K27me3 augments the expression of inflammation genes and the progression of DN.

**FIGURE 3:**
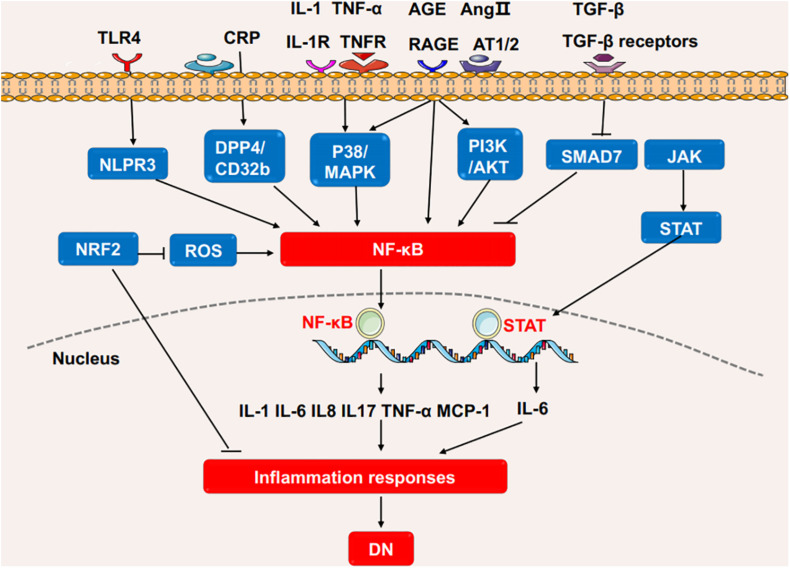
The roles of DNA methylation and histone modification in the DN process. High glucose or stimulating factors cause DNA methylation and histone modification. DNA methylation is mainly regulated by DNMTs. MIC-1 and TIIA inhibits DNA methylation and reduces inflammation in DN. In the processes of histone modification, SET7/9 regulates H3K4 methylation, UTX regulates H3K27 demethylation, HAT promotes histone acetylation, and HDAC4/9 promotes histone deacetylation. The above processes regulate inflammatory genes, in turn affects the inflammatory response in DN. DN, diabetic nephropathy; DNMTs, DNA methyltransferases; UTX, ubiquitously transcribed tetratricopeptide repeat, X chromosome; HAT, histone acetyltransferases; HDAC, histone deacetylase; NF-κB, Nuclear factor-κB; MCP-1, monocyte chemoattractant protein-1; STAT1, Signal transducer and activator of transcription 1.

Similarly, acetylation and deacetylation of histones via histone acetyltransferases (HATs) and histone deacetylase (HDACs) contribute to the pathogenesis of DN. Hyperglycemia promotes chromatin histone acetylation at inflammatory genes promoter regions and enhances inflammatory gene expressions *in vivo* (Miao et al., 2004). Levels of H3K9ac, H3K9ac/S10p, H3K18ac, H3K23ac and H3K56ac are increased in the kidneys of db/db mice (Huang et al., 2015). Furthermore, the expression of *Sirt6* (a histone deacetylase) is reduced in podocytes of STZ-induced mice which results high levels of H3K9ac at promoters of *Notch1* and *Notch4*, and exacerbates the inflammation in kidney (Liu et al., 2017). The silencing of HDAC9 attenuates renal injuries as demonstrated by the decrease in glomerulosclerosis, inflammatory cytokines, and alteration of podocyte apoptosis (Liu et al., 2016). The elevated HDAC4 in diabetic kidney exacerbates inflammation via suppressing STAT1 signaling and the silencing of HDAC4 is associated with the decreases of cytokines (TNF-α, TGF-β, IL-8, MCP-1) (Wang et al., 2014). The roles of DNA methylation and histone modification in the DN process are briefly shown in Figure 3.

### Non-coding RNAs Involved in Inflammation of DN

Non-coding RNAs (ncRNAs) commonly include transfer RNA, ribosomal RNA, long ncRNA (lncRNA), small ncRNA (e.g., microRNA, piRNAs, snoRNA, snRNA, exRNA) and circular RNA (circRNA) (Storz, 2002; Yang, 2015). Roles of microRNA (miRNA), lncRNA and circRNA in DN have been recently studied (Loganathan et al., 2020; Zhou et al., 2021). MiRNA is the best characterized non-coding RNA for transcriptional gene regulation by targeting the 3′-UTR of a specific mRNA. Typically, miRNAs exert their inhibitory actions on the gene via RNA silencing and translational repression (Wilczynska and Bushell, 2015).

MiRNAs play significant roles in regulating inflammation in DN (Zhou et al., 2021). Recent studies involving models of DN podocytes have found that downregulation of the miR-17∼92 cluster ameliorates inflammation and podocyte injury by targeting ABCA1 (ATP-binding cassette transporter A1) (Fan et al., 2020). Similarly, the inhibition of miR-21-5p in a macrophage-derived extracellular vesicle model could also exert podocyte protective effect by the restraint of inflammasome activation (Ding et al., 2020). Moreover, miRNAs are also found to regulate inflammation in renal tubular epithelial cells. The overexpression of miR-199a-3p improves the injury in high glucose induced HK-2 cell damage model, following with decreased IL-1, IL-6 and TNF-α level, which is also consistent with the clinical finding that miR-199a-3p is negatively correlated with the progression of DN (Zhang et al., 2020b). The protective effects of miR-199a-3p is via suppressing miR-199a-3p mediated IKKβ/NF-κB pathway (Zhang et al., 2020b). *In vitro* experiments, the overexpression of miR-26a-5p significantly inhibits the bovine serum albumin (BSA)-induced IL-6 and TNF-α expression in HK2 cells while the inhibition of miR-26a-5p promotes the expression of inflammatory cytokines (Li et al., 2020c). MiR-26a-5p is also found to activate NF-κB pathway by targeting on *CHAC1* and *TLR4* genes (Zhong et al., 2018; Li et al., 2020c). MiR-155 and miR-146a have also been found to be correlated with renal damage, possibly due to the increased expression of TNF-α, TGF-β1, and NF-κB, and their roles in inflammation-mediated glomerular endothelial damage (Huang et al., 2014). Moreover, miRNAs regulate inflammation by modulating macrophage polarization. As mentioned before, macrophage M1 polarization act as an inflammation driver. In miR-146a deficiency diabetic mice, the expression of M1 markers is increased while the M2 response is diminished which is in accordance with the upregulated pro-inflammatory cytokines, suggesting the anti-inflammatory proporities of miR-146a (Bhatt et al., 2016). M2 macrophages ameliorate podocyte injury is related to miR-25-3p (Huang et al., 2020). It is found that autophagy deficiency in diabetic mice increases macrophage infiltration in proximal tubules (Ma et al., 2020), and the induction of miR-214 enhances the autophagy impairment, thus aggravating renal inflammation (Li et al., 2011). MiR-214 in monocytes is upregulated by AGEs, which in turn impairs the expression of the phosphatase and tensin homolog (PTEN) and delays spontaneous apoptosis of monocytes (Li et al., 2011). Additionally, miR-27a is downregulated by an adipokine, omentin-1, which alleviates inflammation and OS by directly targeting the 3′-UTR of *Nrf2* (Song et al., 2018). MiR-29b attenuates podocyte injury by targeting the 3′-UTR of HADC4 in DN (Gondaliya et al., 2020). MiR-125b has been found to inhibit the chromatin histone H3K9 methyltransferase to regulate inflammatory genes in diabetic mice (Villeneuve et al., 2010). Hyperglycemia induces miR-101b, which targets the EZH2, leading to mesangial dysfunction in DN (Jia et al., 2019).

Moreover, accumulating evidence shows that a lot of miRNAs are involved in the regulation of inflammation in DN as shown in Table 1.

**TABLE 1:** The target genes and potential mechanisms of miRNAs associated with inflammation in DN.

miR	Targeted Genes/Pathway	Inflammation Pathway/Related mediator	Sample	Model	Effect on Inflammation	References
miR-15b-5p	*Sema3A*	IL-1β, TNF-α, and IL-6.	Cells	Mouse podocytes	Alleviate	Fu et al., 2019b
miR-21	*Timp3*	Podocyte apoptosis	Rats	STZ-induced DN rats	Promote	Chen et al., 2018
			Cells	HG-treated podocytes		
miR-21	N/D	NF-κB	Mice	Db/db mice	Promote	Zhong et al., 2013
miR-29	*KEAP1*	SIRT1/NF-κB/microR-29/Keap1	Rats	STZ-induced DN rats	Alleviat	Zhou et al., 2015
			Cells	HG-induced injury in HK-2 cells		
miR-29b	*Sp1*	TNF-α, MCP-1/NF-κB	Mice	Db/db mice	Alleviate	Chen et al., 2014b
miR-31	N/D	The recruitment of leukocytes to vascular walls	Serum	DN patients	Alleviate	Rovira-Llopis et al., 2018
miR-126	*Vegf*	PI3K/AKT/mTOR IL-1β, IL-6, IL-18 and TNF-α	Rats	STZ-induced DN rats	Alleviate	Lou et al., 2020
			Cells	NRK52E		
miR-133	N/D	MAPK/ERK	Rats Cells	STZ-induced DN rats HG-induced injury in HK-2 cells	Promote	Shao et al., 2019
miR-140-5p	N/D	TLR4, NF-κB	Tissue	Kidney tissues from DN patients	Alleviate	Lou et al., 2020
			Cells	HG-induced injury in HK-2 cells		
miR-217	N/D	SIRT1/VEGF/HIF-1α	Serum	DN patients	Promote	Shao et al., 2017
miR-217	N/D	N/A	Cells	Rat glomerular mesangial cells	Promote	Shao et al., 2016
miR-218	N/D	NF-κB	Rats	Rat streptozotocin-induced model of DN	Alleviate	Li et al., 2020b
miR-218	*DACH1*	TNF-α and IL-1β	Cells	HG-induced injury in HK-2 cells	Peomote	Zhang et al., 2020c
miR-218	*GPRC5A*	N/A	Cells	HG-induced injury in HK-2 cells	Promote	Su et al., 2020
miR-325-3p	*CCL19*	N/A	Cells	HK-2 and human MC cells	Promote	Sun et al., 2020a
miR-328-3p	*Tlr4*	TLR4, NF-κB	Cells	MP5 cells	Alleviate	Duan et al., 2020
miR-34b	N/D	IL-6R/JAK2/STAT3	Cells	HG-induced HK-2 cells	Promote	Lv et al., 2019
miR-451	*Lmp7*	NF-κB	Mice	Db/db mice	Alleviate	Sun et al., 2016
			Cells	HG-induced MCs		
miR-485	*NOX5*	N/A	Cells	Human MCs	Alleviate	Wu et al., 2020
miR-544	*Fasn*	NF-κB	Mice	Db/db mice	Alleviate	Sun et al., 2020b
miR-770-5p	*Timp3*	IL-1β, TNF-α	Cells	HG-induced mouse podocytes	Promote	Wang and Li, 2020
miR-874	N/D	TLR4	Rats	STZ-induced DN rats	Alleviate	Yao et al., 2019
			Cells	HG-induced podocytes		

*N/D, not determined; N/A, not available; IL, interleukin; TNF, tumor necrosis factor; DN, diabetic nephropathy; STZ, streptozotocin; HG, High glucose; NF-κB, Nuclear factor-κB; SIRT1, Silent information regulator 1; KEAP1, Kelch-like ECH-associated protein 1; HK-2, human kidney 2; Sp1, specificity protein 1; MCP-1, monocyte chemoattractant protein-1; VEGF, vascular endothelial growth factor; HIF-1α, hypoxia-inducible factor-1α; NRK52E, rat kidney tubular epithelial cells; MCs, mesangial cells; TLR4, Toll-like receptors 4; MAPK/ERK, mitogen-activated protein kinase/extracellular signal-regulated kinase; JAK2, Janus kinase 2; STAT3, Signal transducer and activator of transcription 3.*

LncRNAs also contribute to the development and progression of DN. LncRNA myocardial infarction associated transcript (MIAT) promotes hyperglycemia-induced podocyte inflammation by sponging miR-130a-3p and the regulation of TLR4 (Zhang et al., 2020a). LncRNA 4930556M19Rik has been found to protect against HG-induced podocyte damage by downregulation miR-27a-3p (Fan and Zhang, 2020). Macrophage-specific lncRNA_7949 mediates macrophage-induced kidney inflammation by the controlling of MCP-1 transcription through TLR4/NF-κB pathway (Lv et al., 2015). TGF-β/Smad3 transits the miRNA profile and promotes renal diseases via regulating transcriptional levels of non-coding RNAs. SMAD3-dependent lncRNAs have been recently uncovered in kidney diseases (Tang et al., 2018a, 2020a). LncRNA Erbb4-IR is responsible for TGF-β/Smad3-regulated renal fibrosis by inhibiting SMAD7 (Feng et al., 2018). It has been reported that lncRNA Erbb4-IR enhances diabetic kidney injury by mediating miR-29b in db/db Mice. Deletion of SMAD3 could down-regulate the lncRNA Erbb4-IR transcription, and therefore protect against renal injury in db/db mice (Sun et al., 2018). LRNA9884, a novel SMAD3-dependent lncRNA, is not only involved into NF-κB-mediated inflammatory responses by activation of macrophage migration inhibitory factor (MIF) in AKI, but also enhances diabetic renal injury via promoting MCP-1-dependent renal inflammation in db/db mice (Zhang et al., 2019b, 2020d; Xu et al., 2020a). The lncRNAs involved in the inflammation of DN are shown in Table 2.

**TABLE 2:** The target genes and potential mechanisms of lncRNAs associated with inflammation in DN.

lncRNA	Targeted Axis/Inflammation Pathway	Sample	Model	Effect on Inflammation	References
XIST	miR-485/*PSMB8*	Human	DN patients	Promote	Wang, 2020
		Cells	Human MCs		
RPPH1	*Gal-3/Mek/Erk*	Mice	Db/db mice	Promote	Zhang et al., 2019a
		Cells	HG-induced MCs		
NEAT1	miR-34c/NLRP3- CASPASE-1-IL-1β	Rats	STZ-induced DN rats	Alleviate	Zhan et al., 2020
		Cells	HBZY-1		
MEG3	miR-181a/*Egr-1*/TLR4 pathway	Rats	DN rat models	Promote	Zha et al., 2019
KCNQ1OT1	miR-506-3p/NLRP3-CASPASE-1-IL-1β	Cells	HG-induced HK-2 cells	Promote	Li et al., 2017
MALAT1	miR-23c/NLRP3-CASPASE-1-IL-1β	Rats Cells	STZ-induced DN rats HG-induced HK-2 cells	Promote	Li et al., 2017
Gm4419	NF-κB/NLRP3 inflammasome	Cells	HG-induced MCs	Promote	Yi et al., 2017
NON-HSAG053901	*Egr-1/TGF-*β	Mice Cells	STZ-induced mice Mesangial cells	Promote	Peng et al., 2019
HOTTIP	miR-455-3p/*WNT-2B*	Cells	HG-inducedSV40-MES13 cells and HK-2 cells	Promote	Zhu et al., 2019
GAS5	miR-452-5p/NLRP3- CASPASE-1-IL-1β	Cells	HK-2 cells	Alleviate	Xie et al., 2019
UCA1	miRNA-206	Rats	DN rat models	Alleviate	Yu et al., 2019
		Cells	HK-2 cells		
LRNA9884	*Mcp-1/Smad3*	Mice	Db/db	Promote	Zhang et al., 2019b
		Cells	Mouse tubular epithelial cells		

*PSMB8, proteasome subunit beta type-8; DN, diabetic nephropathy; MCs, mesangial cells; HG, high glucose; STZ, streptozotocin; NLRP3, NOD-like receptor protein 3; IL, interleukin; STZ, streptozotocin; HBZY-1, Rat glomerular mesangial cell line; TLR4, Toll-like receptors 4; Egr-1, Early growth response protein 1; HK-2, human kidney tubular epithelial cell 2; WNT-2B, a protein of the Wnt signaling pathway.*

CircRNAs regulate gene expressions by acting as sponges of miRNA (Kristensen et al., 2019), and play an important role in renal diseases (Jin et al., 2020). As a sponge of miR-135a, circRNA_010383 is markedly decreased in the kidney of db/db mice and HG-induced kidney resident cells, and overexpression of circRNA_010383 in kidney protects kidney from proteinuria and fibrosis in DN (Peng et al., 2021). CircLRP6, as a sponge of miR-205, activates TLR4/NF-κB pathway and induces inflammation in high glucose treated mesangial cells (Chen et al., 2019a). CircACTR2 induces inflammation and pyroptosis in high glucose treated renal tubular cells (Wen et al., 2020). Circ_0003928 attenuates the high glucose-induced inflammation in HK-2 cells by targeting miR-151-3p/Anxa2 (An et al., 2020). CircWBSCR17 aggravates inflammation and fibrosis in high glucose-induced HK-2 cells via miR-185-5p/SOX6 axis (Li et al., 2020a). Circ0000285 enhances inflammation via sponging miR-654-3p in high glucose treated podocytes and diabetic mouse kidney (Yao et al., 2020).

## Discussion

The current evidence reveals epigenetics (methylation, acetylation, and non-coding RNA modification) modulate inflammation via intrinsic cells, immune cells, and numerous inflammatory pathways in the development of DN. Persistent inflammation in DN promotes the renal fibrosis, thus resulting in CKD and even end-stage renal disease (Tang et al., 2020a). Anti-inflammatory therapy has long been considered to have enormous benefits for either the alleviation or the prevention of DN (Barutta et al., 2015). In this review, we summarized the evidence linking epigenetic modifications and inflammation in DN. Thus, it may be an effective approach to target these modifications for DN treatment. As for histone modification, the inhibition of HATs/HDACs provides as a class of new agents or therapeutic targets for the treatment of DN. Most of agents are non-selective inhibitors hindering the clinical application (Wang et al., 2014). Valproic acid is a specific HDAC1 inhibitor, which attenuates proteinuria, fibrosis, and inflammatory effects and even acute pancreatitis (Van Beneden et al., 2011; Jain et al., 2019). However, effects of specific HDAC inhibitors for DN remain largely unexplored.

LncRNAs have been considered the novel markers as well as the potential therapeutic targets, and novel drug delivery vehicles (e.g., exosome-ncRNAs). Metformin has been found to protect against inflammation and ECM accumulation in mesangial cells via the H19/miR-143-3p/TGF-β1 axis, suggesting that the H19/miR-143-3p/TGF-β1 axis could be a potential therapeutic target for the management of DN (Xu et al., 2020b). The competing endogenous RNA (ceRNA) network analysis on human miRNA indicates that RP11-363E7.4/TTN-AS1/HOTAIRM1-hsa-miR-106b-5p-PTGER3 and LINC00960-hsa-miR-1237-3p-MMP-2 interaction pairs are significant in diabetic kidney (Yu et al., 2021). Drugs such as iloprost, treprostinil, and captopril that target PTGER3 and MMP-2 might benefit patients with DN (Yu et al., 2021).

Intriguingly, several studies show miRNA-192 is upregulated in diabetic patients with microalbuminuria, but downregulated in macroalbumnuria compared to normalbuminuria (Krupa et al., 2010; Jia et al., 2016). However, another study shows that miR-192 is increased in DN patients with over proteinuria (ACT >300 mg/g) compared to microalbuminuria (Chien et al., 2016). These studies indicate miRNA-mediated epigenetic modifications may have various roles in different stages of a disease.

Besides DNA methylation, histone modification and non-coding RNA, RNA methylation plays an important role in the mRNA post-translational modification. For example, N6-methyladenosine (m^6^A) methylation is the most chemically modified form of eukaryotic messenger RNA (mRNA) which modifies the adenosine at the 3’-UTR and the stop codon of a mRNA (Fu et al., 2014; Roundtree et al., 2017). Roles of epigenetic modifications are not fully elucidated. Recently, single nucleus ATCT-seq integrated with snRNA-seq has been used to detect the cell-type-specific chromatin accessibility which enable to deep understanding of cell heterogeneity in kidney (Bansal et al., 2020; Muto et al., 2021). It may provide a new approach to understand the epigentic modifications in DN.

Collectively, further studies are warranted to reveal the precise regulatory mechanisms in the different stages of DN as well as potential therapeutic targets and diagnostic biomarkers for DN.

## Author Contributions

H-YC and X-MM conceived, revised and edited the manuscript. B-YS and S-FZ collected studies and drafted the manuscript. H-DL assisted in data extraction and revised the manuscript. All authors approved the final version of the manuscript for publication.

## Conflict of Interest

The authors declare that the research was conducted in the absence of any commercial or financial relationships that could be construed as a potential conflict of interest.

## References

[B1] Adhikary L. Chow F. Nikolic-Paterson D. J. Stambe C. Dowling J. Atkins R. C. (2004). Abnormal p38 mitogen-activated protein kinase signalling in human and experimental diabetic nephropathy. *Diabetologia* 47 1210–1222. 10.1007/s00125-004-1437-0 15232685

[B2] Ahmad A. Biersack B. Li Y. Kong D. Bao B. Schobert R. (2013). Targeted regulation of PI3K/Akt/mTOR/NF-κB signaling by indole compounds and their derivatives: mechanistic details and biological implications for cancer therapy. *Anti Cancer Agents Med. Chem.* 13 1002–1013. 10.2174/18715206113139990078 23272910 PMC3901097

[B3] An L. Ji D. Hu W. Wang J. Jin X. Qu Y. (2020). Interference of Hsa_circ_0003928 alleviates high glucose-induced cell apoptosis and inflammation in HK-2 cells via miR-151-3p/Anxa2. *Diabetes Metab Syndr Obes* 13 3157–3168. 10.2147/dmso.s265543 32982348 PMC7494388

[B4] Awad A. S. You H. Gao T. Cooper T. K. Nedospasov S. A. Vacher J. (2015). Macrophage-derived tumor necrosis factor-α mediates diabetic renal injury. *Kidney Int.* 88 722–733. 10.1038/ki.2015.162 26061548 PMC4589442

[B5] Bansal A. Balasubramanian S. Dhawan S. Leung A. Chen Z. Natarajan R. (2020). Integrative omics analyses reveal epigenetic memory in diabetic renal cells regulating genes associated with kidney dysfunction. *Diabetes* 69 2490–2502. 10.2337/db20-0382 32747424 PMC7576555

[B6] Barski A. Cuddapah S. Cui K. Roh T.-Y. Schones D. E. Wang Z. (2007). High-resolution profiling of histone methylations in the human genome. *Cell* 129 823–837. 10.1016/j.cell.2007.05.009 17512414

[B7] Barutta F. Bruno G. Grimaldi S. Gruden G. (2015). Inflammation in diabetic nephropathy: moving toward clinical biomarkers and targets for treatment. *Endocrine* 48 730–742. 10.1007/s12020-014-0437-1 25273317

[B8] Berthier C. C. Zhang H. Schin M. Henger A. Nelson R. G. Yee B. (2009). Enhanced expression of a janus kinase–signal transducer and activator of transcription pathway members in human diabetic nephropathy. *Diabetes* 58 469–477. 10.2337/db08-1328 19017763 PMC2628622

[B9] Bhatt K. Lanting L. L. Jia Y. Yadav S. Reddy M. A. Magilnick N. (2016). Anti-inflammatory role of microRNA-146a in the pathogenesis of diabetic nephropathy. *J. Am. Soc. Nephrol.* 27 2277–2288. 10.1681/asn.2015010111 26647423 PMC4978034

[B10] Chen B. Li Y. Liu Y. Xu Z. (2019a). circLRP6 regulates high glucose-induced proliferation, oxidative stress, ECM accumulation, and inflammation in mesangial cells. *J. Cell. Physiol.* 234 21249–21259. 10.1002/jcp.28730 31087368

[B11] Chen G. Chen H. Ren S. Xia M. Zhu J. Liu Y. (2019b). Aberrant DNA methylation of mTOR pathway genes promotes inflammatory activation of immune cells in diabetic kidney disease. *Kidney Int.* 96 409–420. 10.1016/j.kint.2019.02.020 31101365

[B12] Chen H. Huang Y. Zhu X. Liu C. Yuan Y. Su H. (2019c). Histone demethylase UTX is a therapeutic target for diabetic kidney disease. *J. Physiol.* 597 1643–1660. 10.1113/jp277367 30516825 PMC6418754

[B13] Chen H. Zhong X. Huang X. R. Meng X. M. You Y. Chung A. C. (2014a). MicroRNA-29b inhibits diabetic nephropathy in db/db mice. *Mol. Ther.* 22 842–853. 10.1038/mt.2013.235 24445937 PMC3982502

[B14] Chen H. Y. Huang X. R. Wang W. Li J. H. Heuchel R. L. Chung A. C. (2011). The protective role of Smad7 in diabetic kidney disease: mechanism and therapeutic potential. *Diabetes* 60 590–601. 10.2337/db10-0403 20980457 PMC3028360

[B15] Chen H. Y. Zhong X. Huang X. R. Meng X. M. You Y. Chung A. C. (2014b). MicroRNA-29b inhibits diabetic nephropathy in db/db mice. *Mol. Ther.* 22 842–853. 24445937 10.1038/mt.2013.235 PMC3982502

[B16] Chen J. Guo Y. Zeng W. Huang L. Pang Q. Nie L. (2014c). ER stress triggers MCP-1 expression through SET7/9-induced histone methylation in the kidneys of db/db mice. *Am. J. Physiol. R. Physiol.* 306 F916–F925. 10.1152/ajprenal.00697.2012 24452638

[B17] Chen J. Saxena G. Mungrue I. N. Lusis A. J. Shalev A. (2008). Thioredoxin-interacting protein: a critical link between glucose toxicity and β-cell apoptosis. *Diabetes* 57 938–944. 10.2337/db07-0715 18171713 PMC3618659

[B18] Chen X. Zhao L. Xing Y. Lin B. (2018). Down-regulation of microRNA-21 reduces inflammation and podocyte apoptosis in diabetic nephropathy by relieving the repression of TIMP3 expression. *Biomed. Pharmacother.* 108 7–14. 10.1016/j.biopha.2018.09.007 30212710

[B19] Cheng D. Gao L. Su S. Sargsyan D. Wu R. Raskin I. (2019). Moringa isothiocyanate activates Nrf2: potential role in diabetic nephropathy. *AAPS J.* 21:31. 10.1208/s12248-019-0301-6 PMC6647035 30783799

[B20] Cheng X. Collins R. E. Zhang X. (2005). Structural and sequence motifs of protein (histone) methylation enzymes. *Annu. Rev. Biophys. Biomol. Struct.* 34 267–294. 10.1146/annurev.biophys.34.040204.144452 15869391 PMC2733851

[B21] Chi K. Geng X. Liu C. Cai G. Hong Q. (2020). Research progress on the role of inflammasomes in kidney disease. *Med. Inf.* 2020:8032797. 10.1155/2020/8032797 PMC7204206 32410864

[B22] Chien H. Y. Chen C. Y. Chiu Y. H. Lin Y. C. Li W. C. (2016). Differential microRNA profiles predict diabetic nephropathy progression in taiwan. *Int. J. Med. Sci.* 13 457–465. 10.7150/ijms.15548 27279796 PMC4893561

[B23] Choi H. J. Park J. H. Park M. Won H. Y. Joo H. S. Lee C. H. (2015). UTX inhibits EMT-induced breast CSC properties by epigenetic repression of EMT genes in cooperation with LSD 1 and HDAC 1. *EMBO Rep.* 16 1288–1298. 10.15252/embr.201540244 26303947 PMC4766458

[B24] Chow F. Nikolic-Paterson D. J. Ozols E. Atkins R. C. Rollin B. Tesch G. H. (2006). Monocyte chemoattractant protein-1 promotes the development of diabetic renal injury in streptozotocin-treated mice. *Kidney Int.* 69 73–80. 10.1038/sj.ki.5000014 16374426

[B25] Chow F. Ozols E. Nikolic-Paterson D. J. Atkins R. C. Tesch G. H. (2004). Macrophages in mouse type 2 diabetic nephropathy: correlation with diabetic state and progressive renal injury. *Kidney Int.* 65 116–128. 10.1111/j.1523-1755.2004.00367.x 14675042

[B26] Chung A. C. Huang X. R. Zhou L. Heuchel R. Lai K. N. Lan H. Y. (2009). Disruption of the Smad7 gene promotes renal fibrosis and inflammation in unilateral ureteral obstruction (UUO) in mice. *Nephrol. Dialysis Transplant.* 24 1443–1454. 10.1093/ndt/gfn699 19096081

[B27] Cortvrindt C. Speeckaert R. Moerman A. Delanghe J. R. Speeckaert M. M. (2017). The role of interleukin-17A in the pathogenesis of kidney diseases. *Pathology* 49 247–258. 10.1016/j.pathol.2017.01.003 28291548

[B28] De Marinis Y. Cai M. Bompada P. Atac D. Kotova O. Johansson M. E. (2016). Epigenetic regulation of the thioredoxin-interacting protein (TXNIP) gene by hyperglycemia in kidney. *Kidney Int.* 89 342–353. 10.1016/j.kint.2015.12.018 26806835

[B29] De Santa F. Totaro M. G. Prosperini E. Notarbartolo S. Testa G. Natoli G. (2007). The histone H3 lysine-27 demethylase Jmjd3 links inflammation to inhibition of polycomb-mediated gene silencing. *Cell* 130 1083–1094. 10.1016/j.cell.2007.08.019 17825402

[B30] Ding X. Jing N. Shen A. Guo F. Song Y. Pan M. (2020). MiR-21-5p in macrophage-derived extracellular vesicles affects podocyte pyroptosis in diabetic nephropathy by regulating A20. *J. Endocrinol. Invest.* Epub ahead of print, 10.1007/s40618-020-01401-7 32930981

[B31] Duan Y. Luo Q. Wang Y. Ma Y. Chen F. Zhu X. (2020). Adipose mesenchymal stem cell-derived extracellular vesicles containing microRNA-26a-5p target TLR4 and protect against diabetic nephropathy. *J. Biol. Chem.* 295 12868–12884. 10.1074/jbc.ra120.012522 32580945 PMC7489897

[B32] Eller K. Kirsch A. Wolf A. M. Sopper S. Tagwerker A. Stanzl U. (2011). Potential role of regulatory T cells in reversing obesity-linked insulin resistance and diabetic nephropathy. *Diabetes* 60 2954–2962. 10.2337/db11-0358 21911743 PMC3198056

[B33] Elliott M. J. Maini R. N. Feldmann M. Kalden J. R. Antoni C. Smolen J. S. (1994). Randomised double-blind comparison of chimeric monoclonal antibody to tumour necrosis factor α (cA2) versus placebo in rheumatoid arthritis. *Lancet* 344 1105–1110. 10.1016/s0140-6736(94)90628-9 7934491

[B34] El-Osta A. Brasacchio D. Yao D. Pocai A. Jones P. L. Roeder R. G. (2008). Transient high glucose causes persistent epigenetic changes and altered gene expression during subsequent normoglycemia. *J. Exp. Med.* 205 2409–2417. 10.1084/jem.20081188 18809715 PMC2556800

[B35] Fan H. Zhang W. (2020). Overexpression of Linc 4930556M19Rik Suppresses High Glucose-Triggered Podocyte Apoptosis, Fibrosis and Inflammation via the miR-27a-3p/Metalloproteinase 3 (TIMP3) Axis in Diabetic Nephropathy. *Med. Sci. Monit. Int. Med. J. Exp. Clin. Res.* 26:e925361. 10.12659/MSM.925361 PMC7500124 32896839

[B36] Fan X. Hao Z. Li Z. Wang X. Wang J. (2020). Inhibition of miR-17~92 cluster ameliorates high glucose-induced podocyte damage. *Med. Inf.* 2020:6126490. 10.1155/2020/6126490 PMC7391105 32774146

[B37] Feng M. Tang P. M.-K. Huang X.-R. Sun S.-F. You Y.-K. Xiao J. (2018). TGF-β mediates renal fibrosis via the Smad3-Erbb4-IR long noncoding RNA axis. *Mol. Ther.* 26 148–161. 10.1016/j.ymthe.2017.09.024 29102563 PMC5763082

[B38] Fu J. Akat K. M. Sun Z. G. Zhang W. J. Schlondorff D. Liu Z. H. (2019a). Single-cell RNA profiling of glomerular cells shows dynamic changes in experimental diabetic kidney disease. *J. Am. Soc. Nephrol.* 30 533–545. 10.1681/asn.2018090896 30846559 PMC6442341

[B39] Fu Y. Dominissini D. Rechavi G. He C. (2014). Gene expression regulation mediated through reversible m 6 A RNA methylation. *Nat. Rev. Gene.* 15 293–306. 10.1038/nrg3724 24662220

[B40] Fu Y. Wang C. Zhang D. Chu X. Zhang Y. Li J. (2019b). miR-15b-5p ameliorated high glucose-induced podocyte injury through repressing apoptosis, oxidative stress, and inflammatory responses by targeting Sema3A. *J. Cell. Physiol.* 234 20869–20878. 10.1002/jcp.28691 31025335

[B41] GBD. (2018). global, regional, and national incidence, prevalence, and years lived with disability for 354 diseases and injuries for 195 countries and territories, 1990-2017: a systematic analysis for the global burden of disease study 2017. *Lancet* 392 1789–1858. 30496104 10.1016/S0140-6736(18)32279-7 PMC6227754

[B42] Goldberg A. D. Allis C. D. Bernstein E. (2007). Epigenetics: a landscape takes shape. *Cell* 128 635–638. 10.1016/j.cell.2007.02.006 17320500

[B43] Gondaliya P. A. Jash K. Tekade R. K. Srivastava A. Kalia K. (2020). miR-29b attenuates histone deacetylase-4 mediated podocyte dysfunction and renal fibrosis in diabetic nephropathy. *J. Diabetes Metab Dis.* 19 13–27. 10.1007/s40200-019-00469-0 32550152 PMC7270417

[B44] Grabias B. M. Konstantopoulos K. (2014). The physical basis of renal fibrosis: effects of altered hydrodynamic forces on kidney homeostasis. *Am. J. Physiol. R. Physiol.* 306 F473–F485. 10.1152/ajprenal.00503.2013 24352503

[B45] Heintzman N. D. Hon G. C. Hawkins R. D. Kheradpour P. Stark A. Harp L. F. (2009). Histone modifications at human enhancers reflect global cell-type-specific gene expression. *Nature* 459 108–112. 10.1038/nature07829 19295514 PMC2910248

[B46] Hickey F. B. Martin F. (2013). Diabetic kidney disease and immune modulation. *Curr. Opin. Pharmacol.* 13 602–612. 10.1016/j.coph.2013.05.002 23721739

[B47] Hong J. N. Li W. W. Wang L. L. Guo H. Jiang Y. Gao Y. J. (2017). Jiangtang decoction ameliorate diabetic nephropathy through the regulation of PI3K/Akt-mediated NF-κB pathways in KK-Ay mice. *Chin. Med.* 12 1–16. 28529539 10.1186/s13020-017-0134-0 PMC5437490

[B48] Hotamisligil G. S. (2017). Inflammation, metaflammation and immunometabolic disorders. *Nature* 542 177–185. 10.1038/nature21363 28179656

[B49] Hsieh C. L. (1999). In vivo activity of murine de novo methyltransferases, Dnmt3a and Dnmt3b. *Mol. Cell. Biol.* 19 8211–8218. 10.1128/mcb.19.12.8211 10567546 PMC84905

[B50] Huang H. Liu H. Tang J. Xu W. Gan H. Fan Q. (2020). M2 macrophage-derived exosomal miR-25-3p improves high glucose-induced podocytes injury through activation autophagy via inhibiting DUSP1 expression. *IUBMB Life* 72 2651–2662. 10.1002/iub.2393 33107695

[B51] Huang J. Wan D. Li J. Chen H. Huang K. Zheng L. (2015). Histone acetyltransferase PCAF regulates inflammatory molecules in the development of renal injury. *Epigenetics* 10 62–72. 10.4161/15592294.2014.990780 25496441 PMC4622516

[B52] Huang Y. Liu Y. Li L. Su B. Yang L. Fan W. (2014). Involvement of inflammation-related miR-155 and miR-146a in diabetic nephropathy: implications for glomerular endothelial injury. *BMC Nephrol.* 15:142. 10.1186/1471-2369-15-142 PMC4236663 25182190

[B53] International Diabetes Federation (2019). *IDF DIABETES ATLAS*, 9th Edn. Available online at: https://diabetesatlas.org/en/ (accessed October 10, 2020).

[B54] Jain A. Haque I. Tayal V. Roy V. (2019). Valproic acid-induced acute pancreatitis. *Indian J. Psychiatry* 61 421–422. 10.4103/psychiatry.indianjpsychiatry_383_18 31391650 PMC6657547

[B55] Jia Y. Guan M. Zheng Z. Zhang Q. Tang C. Xu W. (2016). miRNAs in urine extracellular vesicles as predictors of early-stage diabetic nephropathy. *J. Diabetes Res.* 2016:7932765. 10.1155/2016/7932765 26942205 PMC4749815

[B56] Jia Y. Reddy M. A. Das S. Oh H. J. Abdollahi M. Yuan H. (2019). Dysregulation of histone H3 lysine 27 trimethylation in transforming growth factor-β1–induced gene expression in mesangial cells and diabetic kidney. *J. Biol. Chem.* 294 12695–12707. 10.1074/jbc.ra119.007575 31266808 PMC6709639

[B57] Jin J. Sun H. Shi C. Yang H. Wu Y. Li W. (2020). Circular RNA in renal diseases. *J. Cell. Mol. Med.* 24 6523–6533. 32333642 10.1111/jcmm.15295 PMC7299708

[B58] Kato M. Natarajan R. (2019). Epigenetics and epigenomics in diabetic kidney disease and metabolic memory. *Nat. Rev. Nephrol.* 15 327–345. 10.1038/s41581-019-0135-6 30894700 PMC6889804

[B59] Keating S. T. El-Osta A. (2013). Glycemic memories and the epigenetic component of diabetic nephropathy. *Curr. Diabetes Rep.* 13 574–581. 10.1007/s11892-013-0383-y 23639991

[B60] Klessens C. Q. Zandbergen M. Wolterbeek R. Bruijn J. A. Rabelink T. J. Bajema I. M. (2017). Macrophages in diabetic nephropathy in patients with type 2 diabetes. *Nephrol. Dialysis Trans.* 32 1322–1329. 10.1093/ndt/gfw260 27416772

[B61] Kouzarides T. (2007). Chromatin modifications and their function. *Cell* 128 693–705. 10.1016/j.cell.2007.02.005 17320507

[B62] Kristensen L. S. Andersen M. S. Stagsted L. V. W. Ebbesen K. K. Hansen T. B. Kjems J. (2019). The biogenesis, biology and characterization of circular RNAs. *Nat. Rev. Gene.* 20 675–691. 10.1038/s41576-019-0158-7 31395983

[B63] Krupa A. Jenkins R. Luo D. D. Lewis A. Phillips A. Fraser D. (2010). Loss of MicroRNA-192 promotes fibrogenesis in diabetic nephropathy. *J. Am. Soc. Nephrol.* 21 438–447. 10.1681/asn.2009050530 20056746 PMC2831862

[B64] Lampropoulou I. T. Stangou M. Sarafidis P. Gouliovaki A. Giamalis P. Tsouchnikas I. (2020). TNF-α pathway and T-cell immunity are activated early during the development of diabetic nephropathy in type II diabetes mellitus. *Clin. Immunol.* 215:108423. 10.1016/j.clim.2020.108423 32304735

[B65] Lan H. Y. (2012). Transforming growth factor-beta/smad signalling in diabetic nephropathy. *Clin. Exp. Pharmacol. Physiol.* 39 731–738. 10.1111/j.1440-1681.2011.05663.x 22211842

[B66] Larkin B. P. Glastras S. J. Chen H. Pollock C. A. Saad S. (2018). DNA methylation and the potential role of demethylating agents in prevention of progressive chronic kidney disease. *FASEB J.* 32 5215–5226. 10.1096/fj.201800205r 29688808

[B67] Lemos D. R. Mcmurdo M. Karaca G. Wilflingseder J. Leaf I. A. Gupta N. (2018). Interleukin-1β activates a MYC-dependent metabolic switch in kidney stromal cells necessary for progressive tubulointerstitial fibrosis. *J. Am. Soc. Nephrol.* 29 1690–1705. 10.1681/asn.2017121283 29739813 PMC6054344

[B68] Li G. Qin Y. Qin S. Zhou X. Zhao W. Zhang D. (2020a). Circ_WBSCR17 aggravates inflammatory responses and fibrosis by targeting miR-185-5p/SOX6 regulatory axis in high glucose-induced human kidney tubular cells. *Life Sci.* 259:118269. 10.1016/j.lfs.2020.118269 32798559

[B69] Li L. M. Hou D. X. Guo Y. L. Yang J. W. Liu Y. Zhang C. Y. (2011). Role of microRNA-214–targeting phosphatase and tensin homolog in advanced glycation end product-induced apoptosis delay in monocytes. *J. Immunol.* 186 2552–2560. 10.4049/jimmunol.1001633 21228352

[B70] Li M. Guo Q. Cai H. Wang H. Ma Z. Zhang X. (2020b). miR-218 regulates diabetic nephropathy via targeting IKK−β and modulating NK−κB-mediated inflammation. *J. Cell. Physiol.* 235 3362–3371. 10.1002/jcp.29224 31549412 PMC13482667

[B71] Li R. Chung A. C. Yu X. Lan H. Y. (2014). MicroRNAs in diabetic kidney disease. *Int. J. Endocrinol.* 2014:593956. 10.1155/2014/593956 PMC3914440 24550986

[B72] Li S. Jia Y. Xue M. Hu F. Zheng Z. Zhang S. (2020c). Inhibiting Rab27a in renal tubular epithelial cells attenuates the inflammation of diabetic kidney disease through the miR-26a-5p/CHAC1/NF-kB pathway. *Life Sci.* 261:118347. 10.1016/j.lfs.2020.118347 32853650

[B73] Li S. Li W. Wu R. Yin R. Sargsyan D. Raskin I. (2020d). Epigenome and transcriptome study of moringa isothiocyanate in mouse kidney mesangial cells induced by high glucose, a potential model for diabetic-induced nephropathy. *AAPS J.* 22:8. 10.1208/s12248-019-0393-z 31807911

[B74] Li X. Zeng L. Cao C. Lu C. Lian W. Han J. (2017). Long noncoding RNA MALAT1 regulates renal tubular epithelial pyroptosis by modulated miR-23c targeting of ELAVL1 in diabetic nephropathy. *Exp. Cell Res.* 350 327–335. 10.1016/j.yexcr.2016.12.006 27964927

[B75] Li Y. Reddy M. A. Miao F. Shanmugam N. Yee J.-K. Hawkins D. (2008). Role of the histone H3 lysine 4 methyltransferase, SET7/9, in the regulation of NF-κB-dependent inflammatory genes: relevance to diabetes and inflammation. *J. Biol. Chem.* 283 26771–26781. 10.1074/jbc.m802800200 18650421 PMC2546554

[B76] Lim A. K. (2014). Diabetic nephropathy–complications and treatment. *Int. J. Nephrol. Renovascular Dis.* 7:361. 10.2147/ijnrd.s40172 25342915 PMC4206379

[B77] Lin J. Cheng A. Cheng K. Deng Q. Zhang S. Lan Z. (2020a). New insights into the mechanisms of pyroptosis and implications for diabetic kidney disease. *Int. J. Mol. Sci.* 21:7057. 10.3390/ijms21197057 32992874 PMC7583981

[B78] Lin X. Xu Y. Pan X. Xu J. Ding Y. Sun X. (2020b). Global, regional, and national burden and trend of diabetes in 195 countries and territories: an analysis from 1990 to 2025. *Sci. Rep.* 10:14790. 10.1038/s41598-020-71908-9 PMC7478957 32901098

[B79] Liu F. Chen H. Y. Huang X. R. Chung A. C. Zhou L. Fu P. (2011). C-reactive protein promotes diabetic kidney disease in a mouse model of type 1 diabetes. *Diabetologia* 54 2713–2723. 10.1007/s00125-011-2237-y 21744073

[B80] Liu F. Zong M. Wen X. Li X. Wang J. Wang Y. (2016). Silencing of histone deacetylase 9 expression in podocytes attenuates kidney injury in diabetic nephropathy. *Sci. Rep.* 6:33676. 10.1038/srep33676 PMC5025656 27633396

[B81] Liu M. Liang K. Zhen J. Zhou M. Wang X. Wang Z. (2017). Sirt6 deficiency exacerbates podocyte injury and proteinuria through targeting Notch signaling. *Nat. Commun.* 8 1–15. 10.1016/j.biochi.2014.12.015 28871079 PMC5583183

[B82] Loganathan T. S. Sulaiman S. A. Abdul Murad N. A. Shah S. A. Abdul Gafor A. H. Jamal R. (2020). Interactions among non-coding RNAs in diabetic nephropathy. *Front. Pharmacol.* 11:191. 10.3389/fphar.2020.0019 PMC7062796 32194418

[B83] Lou Z. Li Q. Wang C. Li Y. (2020). The effects of microRNA-126 reduced inflammation and apoptosis of diabetic nephropathy through PI3K/AKT signalling pathway by VEGF. *Arch. Physiol. Biochem.* 25 1–10. 10.1080/13813455.2020.1767146 32449863

[B84] Lv L. Tang P. You Y. K. Huang X. Liu B.-C. Lan H.-Y. (2015). Long noncoding RNA-7949 regulates macrophage activation in renal inflammation via the TLR4/NF-KB pathway. *Hong Kong J. Nephrol.* 2:S76.

[B85] Lv N. Li C. Liu X. Qi C. Wang Z. (2019). miR-34b alleviates high glucose-induced inflammation and apoptosis in human HK-2 cells via IL-6R/JAK2/STAT3 signaling pathway. *Med. Sci. Monitor Int. Med. J. Exp. Clin. Res.* 25:8142. 10.12659/msm.917128 31665127 PMC6842269

[B86] Ma Z. Li L. Livingston M. J. Zhang D. Mi Q. Zhang M. (2020). p53/microRNA-214/ULK1 axis impairs renal tubular autophagy in diabetic kidney disease. *J. Clin. Invest.* 130 5011–5026. 10.1172/jci135536 32804155 PMC7456229

[B87] Majumder S. Thieme K. Batchu S. N. Alghamdi T. A. Bowskill B. B. Kabir M. G. (2018). Shifts in podocyte histone H3K27me3 regulate mouse and human glomerular disease. *J. Clin. Invest.* 128 483–499. 10.1172/jci95946 29227285 PMC5749498

[B88] Meng X. M. Nikolic-Paterson D. J. Lan H. Y. (2014). Inflammatory processes in renal fibrosis. *Nat. Rev. Nephrol.* 10 493–503. 10.1038/nrneph.2014.114 24981817

[B89] Meng X. M. Wang S. Huang X. R. Yang C. Xiao J. Zhang Y. (2016). Inflammatory macrophages can transdifferentiate into myofibroblasts during renal fibrosis. *Cell Death Dis.* 7:e2495. 10.1038/cddis.2016.402 27906172 PMC5261004

[B90] Miao F. Gonzalo I. G. Lanting L. Natarajan R. (2004). In vivo chromatin remodeling events leading to inflammatory gene transcription under diabetic conditions. *J. Biol. Chem.* 279 18091–18097. 10.1074/jbc.m311786200 14976218

[B91] Mikuda N. Kolesnichenko M. Beaudette P. Popp O. Uyar B. Sun W. (2018). The IκB kinase complex is a regulator of mRNA stability. *EMBO J.* 37:e98658. 10.15252/embj.201798658 PMC6293339 30467221

[B92] Moon J. Y. Jeong K. H. Lee T. W. Ihm C. G. Lim S. J. Lee S. H. (2012). Aberrant recruitment and activation of T cells in diabetic nephropathy. *Am. J. Nephrol.* 35 164–174. 10.1159/000334928 22286547

[B93] Moriwaki Y. Inokuchi T. Yamamoto A. Ka T. Tsutsumi Z. Takahashi S. (2007). Effect of TNF-α inhibition on urinary albumin excretion in experimental diabetic rats. *Acta Diabetologica* 44 215–218. 10.1007/s00592-007-0007-6 17767370

[B94] Muto Y. Wilson P. Wu H. Waikar S. Humphreys B. (2021). Single cell transcriptional and chromatin accessibility profiling redefine cellular heterogeneity in the adult human kidney. *Nat. Commun.* 12:2190. 10.1038/s41467-021-22368-w 33850129 PMC8044133

[B95] Navarro-González J. F. Mora-Fernández C. De Fuentes M. M. Chahin J. Méndez M. L. Gallego E. (2015). Effect of pentoxifylline on renal function and urinary albumin excretion in patients with diabetic kidney disease: the PREDIAN trial. *J. Am. Soc. Nephrol.* 26 220–229. 10.1681/asn.2014010012 24970885 PMC4279740

[B96] Newton K. Dixit V. M. (2012). Signaling in innate immunity and inflammation. *Cold Spring Harbor Perspect. Biol.* 4:a006049. 10.1101/cshperspect.a006049 PMC3282411 22296764

[B97] O’shea J. J. Kontzias A. Yamaoka K. Tanaka Y. Laurence A. (2013). Janus kinase inhibitors in autoimmune diseases. *Ann. Rheumatic Dis.* 72:ii111. 10.1136/annrheumdis-2012-202576 23532440 PMC3616338

[B98] Park J. Guan Y. Sheng X. Gluck C. Seasock M. J. Hakimi A. A. (2019). Functional methylome analysis of human diabetic kidney disease. *JCI Insight* 4:e128886. 10.1172/jci.insight.128886 PMC6629092 31167971

[B99] Peng F. Gong W. Li S. Yin B. Zhao C. Liu W. (2021). circRNA_010383 acts as a sponge for miR-135a, and its downregulated expression contributes to renal fibrosis in diabetic nephropathy. *Diabetes* 70 603–615. 10.2337/db20-0203 33472945

[B100] Peng W. F. Huang S. Shen L. S. Tang Y. B. Li H. H. Shi Y. Q. (2019). Long noncoding RNA NONHSAG053901 promotes diabetic nephropathy via stimulating Egr-1/TGF-beta-mediated renal inflammation. *J. Cell. Physiol.* 234 18492–18503. 10.1002/jcp.28485 30927260

[B101] Pergola P. E. Raskin P. Toto R. D. Meyer C. J. Huff J. W. Grossman E. B. (2011). Bardoxolone methyl and kidney function in CKD with type 2 diabetes. *New Engl. J. Med.* 365 327–336. 21699484 10.1056/NEJMoa1105351

[B102] Pina T. Corrales A. Lopez-Mejias R. Armesto S. Gonzalez-Lopez M. A. Gómez-Acebo I. (2016). Anti-tumor necrosis factor-alpha therapy improves endothelial function and arterial stiffness in patients with moderate to severe psoriasis: a 6-month prospective study. *J. Dermatol.* 43 1267–1272. 10.1111/1346-8138.13398 27062420

[B103] Pradeepa M. M. Grimes G. R. Kumar Y. Olley G. Taylor G. C. Schneider R. (2016). Histone H3 globular domain acetylation identifies a new class of enhancers. *Nat. Genet.* 48:681. 10.1038/ng.3550 27089178 PMC4886833

[B104] Reddy M. A. Zhang E. Natarajan R. (2015). Epigenetic mechanisms in diabetic complications and metabolic memory. *Diabetologia* 58 443–455. 10.1007/s00125-014-3462-y 25481708 PMC4324095

[B105] Roundtree I. A. Evans M. E. Pan T. He C. (2017). Dynamic RNA modifications in gene expression regulation. *Cell* 169 1187–1200. 10.1016/j.cell.2017.05.045 28622506 PMC5657247

[B106] Rovira-Llopis S. Escribano-Lopez I. Diaz-Morales N. Iannantuoni F. Lopez-Domenech S. Andújar I. (2018). Downregulation of miR-31 in diabetic nephropathy and its relationship with inflammation. *Cell. Physiol. Biochem.* 50 1005–1014. 10.1159/000494485 30355913

[B107] Ruiz-Ortega M. Rayego-Mateos S. Lamas S. Ortiz A. Rodrigues-Diez R. R. (2020). Targeting the progression of chronic kidney disease. *Nat. Rev. Nephrol.* 16 269–288. 32060481 10.1038/s41581-019-0248-y

[B108] Russo V. E. Martienssen R. A. Riggs A. D. (1996). *Epigenetic Mechanisms of Gene Regulation.* New York, NY: Cold Spring Harbor Laboratory Press.

[B109] Sanz A. B. Ramos A. M. Soler M. J. Sanchez-Niño M. D. Fernandez-Fernandez B. Perez-Gomez M. V. (2019). Advances in understanding the role of angiotensin-regulated proteins in kidney diseases. *Exp. Rev. Proteomics* 16 77–92. 10.1080/14789450.2018.1545577 30412432

[B110] Saraheimo M. Teppo A.-M. Forsblom C. Fagerudd J. Groop P.-H. Group F. S. (2003). Diabetic nephropathy is associated with low-grade inflammation in type 1 diabetic patients. *Diabetologia* 46 1402–1407. 10.1007/s00125-003-1194-5 12928771

[B111] Schena F. P. Gesualdo L. (2005). Pathogenetic mechanisms of diabetic nephropathy. *J. Am. Soc. Nephrol.* 16 S30–S33. 15938030 10.1681/asn.2004110970

[B112] Shahzad K. Bock F. Dong W. Wang H. Kopf S. Kohli S. (2015). Nlrp3-inflammasome activation in non-myeloid-derived cells aggravates diabetic nephropathy. *Kidney Int.* 87 74–84. 10.1038/ki.2014.271 25075770 PMC4284813

[B113] Shang J. Zhang Y. Jiang Y. Li Z. Duan Y. Wang L. (2017). NOD2 promotes endothelial-to-mesenchymal transition of glomerular endothelial cells via MEK/ERK signaling pathway in diabetic nephropathy. *Biochem. Biophys. Res. Commun.* 484 435–441. 10.1016/j.bbrc.2017.01.155 28137583

[B114] Shao X. Kong W. X. Li Y. T. (2019). MiR-133 inhibits kidney injury in rats with diabetic nephropathy via MAPK/ERK pathway. *Eur. Rev. Med. Pharmacol. Sci.* 23 10957–10963. 31858564 10.26355/eurrev_201912_19799

[B115] Shao Y. Lv C. Wu C. Zhou Y. Wang Q. (2016). Mir-217 promotes inflammation and fibrosis in high glucose cultured rat glomerular mesangial cells via Sirt1/HIF-1α signaling pathway. *Diabetes Metab. Res. Rev.* 32 534–543. 10.1002/dmrr.2788 26891083

[B116] Shao Y. Ren H. Lv C. Ma X. Wu C. Wang Q. (2017). Changes of serum Mir-217 and the correlation with the severity in type 2 diabetes patients with different stages of diabetic kidney disease. *Endocrine* 55 130–138. 10.1007/s12020-016-1069-4 27522360

[B117] Sheng J. Wang L. Tang P. M. Wang H. L. Li J. C. Xu B. H. (2021). Smad3 deficiency promotes beta cell proliferation and function in db/db mice via restoring Pax6 expression. *Theranostics* 11 2845–2859. 10.7150/thno.51857 33456576 PMC7806493

[B118] Song J. Zhang H. Sun Y. Guo R. Zhong D. Xu R. (2018). Omentin-1 protects renal function of mice with type 2 diabetic nephropathy via regulating miR-27a-Nrf2/Keap1 axis. *Biomed. Pharmacother.* 107 440–446. 10.1016/j.biopha.2018.08.002 30103116

[B119] Storz G. (2002). An expanding universe of noncoding RNAs. *Science* 296 1260–1263. 10.1126/science.1072249 12016301

[B120] Su S.-S. Li B.-P. Li C.-L. Xiu F.-R. Wang D.-Y. Zhang F.-R. (2020). Downregulation of MiR-218 can alleviate high-glucose-induced renal proximal tubule injury by targeting GPRC5A. *Biosci. Biotechnol. Biochem.* 84 1123–1130. 10.1080/09168451.2020.1717330 32028854

[B121] Sun H. Tian J. Xian W. Xie T. Yang X. (2015). Pentraxin-3 attenuates renal damage in diabetic nephropathy by promoting M2 macrophage differentiation. *Inflammation* 38 1739–1747. 10.1007/s10753-015-0151-z 25761429

[B122] Sun J. Wang J. Lu W. Xie L. Lv J. Li H. (2020a). MiR-325-3p inhibits renal inflammation and fibrosis by targeting CCL19 in diabetic nephropathy. *Clin. Exp. Pharmacol. Physiol.* 47 1850–1860. 32603491 10.1111/1440-1681.13371

[B123] Sun S. F. Tang P. M. Feng M. Xiao J. Huang X. R. Li P. (2018). Novel lncRNA Erbb4-IR promotes diabetic kidney injury in db/db mice by targeting miR-29b. *Diabetes* 67 731–744. 10.2337/db17-0816 29222368

[B124] Sun T. Liu Y. Liu L. Ma F. (2020b). MicroRNA-544 attenuates diabetic renal injury via suppressing glomerulosclerosis and inflammation by targeting FASN. *Gene* 723:143986. 10.1016/j.gene.2019.143986 31323309

[B125] Sun Y. Peng R. Peng H. Liu H. Wen L. Wu T. (2016). miR-451 suppresses the NF-kappaB-mediated proinflammatory molecules expression through inhibiting LMP7 in diabetic nephropathy. *Mol. Cell. Endocrinol.* 433 75–86. 10.1016/j.mce.2016.06.004 27264074

[B126] Tang P. C.-T. Zhang Y.-Y. Chan M. K.-K. Lam W. W.-Y. Chung J. Y.-F. Kang W. (2020a). The emerging role of innate immunity in chronic kidney diseases. *Int. J. Mol. Sci.* 21:4018. 10.3390/ijms21114018 32512831 PMC7312694

[B127] Tang P. M. K. Nikolic-Paterson D. J. Lan H. Y. (2019). Macrophages: versatile players in renal inflammation and fibrosis. *Nat. Rev. Nephrol.* 15 144–158. 10.1038/s41581-019-0110-2 30692665

[B128] Tang P. M. K. Zhang Y. Y. Hung J. S. C. Chung J. Y. F. Huang X. R. To K. F. (2021). DPP4/CD32b/NF-κB Circuit: a novel druggable target for inhibiting CRP-driven diabetic nephropathy. *Mol. Ther.* 29 365–375. 10.1016/j.ymthe.2020.08.017 32956626 PMC7790911

[B129] Tang P. M. K. Zhang Y. Y. Mak T. S. K. Tang P. C. T. Huang X. R. Lan H. Y. (2018a). Transforming growth factor−β signalling in renal fibrosis: from Smads to non-coding RNAs. *J. Physiol.* 596 3493–3503. 10.1113/jp274492 29781524 PMC6092283

[B130] Tang P. M. K. Zhang Y. Y. Xiao J. Tang P. C. T. Chung J. Y. F. Li J. (2020b). Neural transcription factor Pou4f1 promotes renal fibrosis via macrophage–myofibroblast transition. *Proc. Natl. Acad. Sci.* 117 20741–20752. 10.1073/pnas.1917663117 32788346 PMC7456094

[B131] Tang P. M. K. Zhou S. Li C. J. Liao J. Xiao J. Wang Q. M. (2018b). The proto-oncogene tyrosine protein kinase Src is essential for macrophage-myofibroblast transition during renal scarring. *Kidney Int.* 93 173–187. 10.1016/j.kint.2017.07.026 29042082

[B132] Tang S. C. W. Yiu W. H. (2020). Innate immunity in diabetic kidney disease. *Nat. Rev. Nephrol.* 16 206–222. 10.1038/s41581-019-0234-4 31942046

[B133] Torres Á Muñoz K. Nahuelpán Y. R Saez A.-P. Mendoza P. Jara C. (2020). Intraglomerular monocyte/macrophage infiltration and macrophage–myofibroblast transition during diabetic nephropathy is regulated by the A2B adenosine receptor. *Cells* 9:1051. 10.3390/cells9041051 32340145 PMC7226348

[B134] Van Beneden K. Geers C. Pauwels M. Mannaerts I. Verbeelen D. Van Grunsven L. A. (2011). Valproic acid attenuates proteinuria and kidney injury. *J. Am. Soc. Nephrol.* 22 1863–1875. 10.1681/asn.2010111196 21868496 PMC3279948

[B135] VanderJagt T. A. Neugebauer M. H. Morgan M. Bowden D. W. Shah V. O. (2015). Epigenetic profiles of pre-diabetes transitioning to type 2 diabetes and nephropathy. *World J. Diabetes* 6:1113. 10.4239/wjd.v6.i9.1113 26265998 PMC4530325

[B136] Villeneuve L. M. Kato M. Reddy M. A. Wang M. Lanting L. Natarajan R. (2010). Enhanced levels of microRNA-125b in vascular smooth muscle cells of diabetic db/db mice lead to increased inflammatory gene expression by targeting the histone methyltransferase Suv39h1. *Diabetes* 59 2904–2915. 10.2337/db10-0208 20699419 PMC2963550

[B137] Wada J. Makino H. (2016). Innate immunity in diabetes and diabetic nephropathy. *Nat. Rev. Nephrol.* 12:13. 10.1038/nrneph.2015.175 26568190

[B138] Wang J. Shen X. Liu J. Chen W. Wu F. Wu W. (2020). High glucose mediates NLRP3 inflammasome activation via upregulation of ELF3 expression. *Cell Death Dis.* 11 1–14. 32439949 10.1038/s41419-020-2598-6 PMC7242464

[B139] Wang L. Li H. (2020). MiR-770-5p facilitates podocyte apoptosis and inflammation in diabetic nephropathy by targeting TIMP3. *Biosci. Rep.* 40:BSR20193653. 10.1042/BSR20193653 PMC7189364 32309847

[B140] Wang Q. (2020). XIST silencing alleviated inflammation and mesangial cells proliferation in diabetic nephropathy by sponging miR-485. *Arch. Physiol. Biochem.* 15 1–7. 10.1080/13813455.2020.1789880 32669002

[B141] Wang X. Liu J. Zhen J. Zhang C. Wan Q. Liu G. (2014). Histone deacetylase 4 selectively contributes to podocyte injury in diabetic nephropathy. *Kidney Int.* 86 712–725. 10.1038/ki.2014.111 24717296

[B142] Wang X. Yao B. Wang Y. Fan X. Wang S. Niu A. (2017). Macrophage cyclooxygenase-2 protects against development of diabetic nephropathy. *Diabetes* 66 494–504. 10.2337/db16-0773 27815317 PMC5248989

[B143] Webster L. Abordo E. A. Thornalley P. J. Limb G. A. (1997). Induction of TNFα and IL-1β mRNA in monocytes by methylglyoxal-and advanced glycated endproduct-modified human serum albumin. *Biochem. Soc. Trans.* 25:250S. 10.1042/bst025250s 9191294

[B144] Wen S. Li S. Li L. Fan Q. (2020). circACTR2: a novel mechanism regulating high glucose-induced fibrosis in renal tubular cells via pyroptosis. *Biol. Pharm. Bull.* 43 558–564. 10.1248/bpb.b19-00901 32115515

[B145] Wilczynska A. Bushell M. (2015). The complexity of miRNA-mediated repression. *Cell Death Differ.* 22 22–33. 10.1038/cdd.2014.112 25190144 PMC4262769

[B146] Wilson P. C. Wu H. J. Kirita Y. Uchimura K. Ledru N. Rennke H. G. (2019). The single-cell transcriptomic landscape of early human diabetic nephropathy. *Proc. Natl. Acad. Sci. U.S.A.* 116 19619–19625. 10.1073/pnas.1908706116 31506348 PMC6765272

[B147] Wu C. H. Huang C. M. Lin C. H. Ho Y. S. Chen C. M. Lee H. M. (2002). Advanced glycosylation end products induce NF-κB dependent iNOS expression in RAW 264.7 cells. *Mol. Cell. Endocrinol.* 194 9–17. 10.1016/s0303-7207(02)00212-5 12242023

[B148] Wu J. Lu K. Zhu M. Xie X. Ding Y. Shao X. (2020). miR-485 suppresses inflammation and proliferation of mesangial cells in an in vitro model of diabetic nephropathy by targeting NOX5. *Biochem. Biophys. Res. Commun.* 521 984–990. 10.1016/j.bbrc.2019.11.020 31727371

[B149] Xie C. Wu W. Tang A. Luo N. Tan Y. (2019). lncRNA GAS5/miR-452-5p reduces oxidative stress and pyroptosis of high-glucose-stimulated renal tubular cells. *Diabetes Metab. Syndrome Obes. Targets Ther.* 12:2609. 10.2147/dmso.s228654 31849505 PMC6910862

[B150] Xu B. H. Sheng J. You Y. K. Huang X. R. Ma R. C. W. Wang Q. (2020a). Deletion of Smad3 prevents renal fibrosis and inflammation in type 2 diabetic nephropathy. *Metabolism* 103:154013. 10.1016/j.metabol.2019.154013 31734275

[B151] Xu J. Xiang P. Liu L. Sun J. Ye S. (2020b). Metformin inhibits extracellular matrix accumulation, inflammation and proliferation of mesangial cells in diabetic nephropathy by regulating H19/miR-143-3p/TGF−β1 axis. *J. Pharmacy Pharmacol.* 72 1101–1109. 10.1111/jphp.13280 32391614

[B152] Yagi S. Hirosawa M. Shiota K. (2012). DNA methylation profile: a composer-, conductor-, and player-orchestrated mammalian genome consisting of genes and transposable genetic elements. *J. Reproduct. Deve.* 58 265–273. 10.1262/jrd.2011-030 22790869

[B153] Yang C. Chen X. C. Li Z. H. Wu H. L. Jing K. P. Huang X. R. (2020). SMAD3 promotes autophagy dysregulation by triggering lysosome depletion in tubular epithelial cells in diabetic nephropathy. *Autophagy* Epub ahead of print, 10.1080/15548627.2020.1824694 PMC8496726 33043774

[B154] Yang L. (2015). Splicing noncoding RNAs from the inside out. *Wiley Interdisciplinary Rev. RNA* 6 651–660. 10.1002/wrna.1307 26424453 PMC5054931

[B155] Yao T. Zha D. Gao P. Shui H. Wu X. (2019). MiR-874 alleviates renal injury and inflammatory response in diabetic nephropathy through targeting toll-like receptor-4. *J. Cell. Physiol.* 234 871–879. 10.1002/jcp.26908 30171701

[B156] Yao T. Zha D. Hu C. Wu X. (2020). Circ_0000285 promotes podocyte injury through sponging miR-654-3p and activating MAPK6 in diabetic nephropathy. *Gene* 747:144661. 10.1016/j.gene.2020.144661 32275999

[B157] Yi H. Peng R. Zhang L. Y. Sun Y. Peng H. M. Liu H. D. (2017). LincRNA-Gm4419 knockdown ameliorates NF-kappa B/NLRP3 inflammasome-mediated inflammation in diabetic nephropathy. *Cell Death Dis.* 8:e2583. 10.1038/cddis.2016.451 28151474 PMC5386454

[B158] You H. Gao T. Cooper T. K. Brian Reeves W. Awad A. S. (2013). Macrophages directly mediate diabetic renal injury. *Am. J. Physiol. R. Physiol.* 305 F1719–F1727. 10.1152/ajprenal.00141.2013 PMC3882451 24173355

[B159] Yu R. Zhang Y. Lu Z. Li J. Shi P. Li J. (2019). Long-chain non-coding RNA UCA1 inhibits renal tubular epithelial cell apoptosis by targeting microRNA-206 in diabetic nephropathy. *Arch. Physiol. Biochem.* Epub ahead of print, 10.1080/13813455.2019.1673431 31608712

[B160] Yu Y. Jia Y. Y. Wang M. Mu L. Li H. J. (2021). PTGER3 and MMP-2 play potential roles in diabetic nephropathy via competing endogenous RNA mechanisms. *BMC Nephrol.* 22:27. 10.1186/s12882-020-02194-w PMC7805187 33435900

[B161] Yuan E. F. Yang Y. Cheng L. Deng X. Chen S. M. Zhou X. (2019). Hyperglycemia affects global 5-methylcytosine and 5-hydroxymethylcytosine in blood genomic DNA through upregulation of SIRT6 and TETs. *Clin. Epigenet.* 11 1–9. 10.1186/s13148-019-0660-y PMC6466651 30987683

[B162] Zha F. Qu X. Tang B. Li J. Wang Y. Zheng P. (2019). Long non-coding RNA MEG3 promotes fibrosis and inflammatory response in diabetic nephropathy via miR-181a/Egr-1/TLR4 axis. *Aging (Albany NY)* 11:3716. 10.18632/aging.102011 31195367 PMC6594792

[B163] Zhan J. F. Huang H. W. Huang C. Hu L. L. Xu W. W. (2020). Long non-coding RNA NEAT1 regulates pyroptosis in diabetic nephropathy via mediating the miR-34c/NLRP3 Axis. *Kidney Blood Pres. Res.* 45 589–602. 10.1159/000508372 32721950

[B164] Zhang C. Xiao C. Wang P. Xu W. Zhang A. Li Q. (2014). The alteration of Th1/Th2/Th17/Treg paradigm in patients with type 2 diabetes mellitus: Relationship with diabetic nephropathy. *Hum. Immunol.* 75 289–296. 10.1016/j.humimm.2014.02.007 24530745

[B165] Zhang H. Nair V. Saha J. Atkins K. B. Hodgin J. B. Saunders T. L. (2017). Podocyte-specific JAK2 overexpression worsens diabetic kidney disease in mice. *Kidney Int.* 92 909–921. 10.1016/j.kint.2017.03.027 28554737 PMC5610635

[B166] Zhang M. Zhao S. Xu C. Shen Y. Huang J. Shen S. (2020a). Ablation of lncRNA MIAT mitigates high glucose-stimulated inflammation and apoptosis of podocyte via miR-130a-3p/TLR4 signaling axis. *Biochem. Biophys. Res. Commun.* 533 429–436. 10.1016/j.bbrc.2020.09.034 32972755

[B167] Zhang P. Sun Y. Peng R. Chen W. Fu X. Zhang L. (2019a). Long non-coding RNA Rpph1 promotes inflammation and proliferation of mesangial cells in diabetic nephropathy via an interaction with Gal-3. *Cell Death Dis.* 10 1–16. 10.1038/s41419-019-1765-0 PMC6614467 31285427

[B168] Zhang R. Qin L. Shi J. (2020b). MicroRNA-199a-3p suppresses high glucose-induced apoptosis and inflammation by regulating the IKKβ/NF−κB signaling pathway in renal tubular epithelial cells. *Int. J. Mol. Med.* 46 2161–2171. 10.3892/ijmm.2020.4751 33125105 PMC7595662

[B169] Zhang Y. L. Wang J. M. Yin H. Wang S. B. He C. L. Liu J. (2020c). DACH1, a novel target of miR-218, participates in the regulation of cell viability, apoptosis, inflammatory response, and epithelial-mesenchymal transition process in renal tubule cells treated by high-glucose. *REN Fail* 42 463–473. 10.1080/0886022x.2020.1762647 32408786 PMC7269034

[B170] Zhang Y. Y. Tang P. M. Niu Y. Córdoba G. A. Alexandra C. Huang X. R. (2020d). Long non-coding RNA LRNA9884 promotes acute kidney injury via regulating NF-kB-mediated transcriptional activation of MIF. *Front. Physiol.* 11:1399. 10.3389/fphys.2020.590027 33192605 PMC7658631

[B171] Zhang Y. Y. Tang P. M. K. Tang P. C. T. Xiao J. Huang X. R. Yu C. (2019b). LRNA9884, a novel smad3-dependent long noncoding RNA, promotes diabetic kidney injury in db/db mice via enhancing MCP-1–dependent renal inflammation. *Diabetes* 68 1485–1498. 10.2337/db18-1075 31048367

[B172] Zhong X. Chung A. C. K. Chen H. Dong Y. Meng X. Li R. (2013). miR-21 is a key therapeutic target for renal injury in a mouse model of type 2 diabetes. *Diabetologia* 56 663–674. 10.1007/s00125-012-2804-x 23292313

[B173] Zhong X. Zhang L. Li Y. Li P. Li J. Cheng G. (2018). Kaempferol alleviates ox-LDL-induced apoptosis by up-regulation of miR-26a-5p via inhibiting TLR4/NF-κB pathway in human endothelial cells. *Biomedicine Pharmacother.* 108 1783–1789. 10.1016/j.biopha.2018.09.175 30372882

[B174] Zhou H. Ni W.-J. Meng X.-M. Tang L.-Q. (2021). MicroRNAs as regulators of immune and inflammatory responses: potential therapeutic targets in diabetic nephropathy. *Front. Cell Dev. Biol.* 8:1837. 10.3389/fcell.2020.618536 33569382 PMC7868417

[B175] Zhou L. Xu D. Y. Sha W. G. Shen L. Lu G. Y. Yin X. (2015). High glucose induces renal tubular epithelial injury via Sirt1/NF-kappaB/microR-29/Keap1 signal pathway. *J. Trans. Med.* 13 1–12. 10.1186/s12967-015-0710-y PMC4640239 26552447

[B176] Zhu X. J. Gong Z. Li S. J. Jia H. P. Li D. L. (2019). Long non-coding RNA Hottip modulates high-glucose-induced inflammation and ECM accumulation through miR-455-3p/WNT2B in mouse mesangial cells. *Int. J. Clin. Exp. Pathol.* 12:2435. PMC6949587 31934070

