# Supplementary material for: Epigenetics and Inflammation in Diabetic Nephropathy
Source: Front Physiol. 2021 May 5;12:649587. doi: 10.3389/fphys.2021.649587 (PMC8131683; doi:10.3389/fphys.2021.649587)
Supplement: Supplementary file 1 [file Data_Sheet_1.PDF]

Supplementary file 1. Search strategies

Publications reviewed in the manuscript were retrieved from PubMed with the following search strategies. The information was updated by Feb 20, 2021

Section	Search strategies
Inflammation in the progression of DN	("Inflammation"[MeSH Terms] OR "Inflammation Mediators"[MeSH Terms]) AND (("diabetes complications"[MeSH Terms] AND "kidney diseases"[MeSH Terms]) OR "Diabetic Nephropathies"[MeSH Terms] OR "Diabetic Nephropathy"[All Fields] OR "Diabetic kidney disease"[All Fields])
DNA methylation involved in inflammation of DN	("Inflammation"[MeSH Terms] OR "Inflammation Mediators"[MeSH Terms]) AND (("diabetes complications"[MeSH Terms] AND "kidney diseases"[MeSH Terms]) OR "Diabetic Nephropathies"[MeSH Terms] OR "Diabetic Nephropathy"[All Fields] OR "Diabetic kidney disease"[All Fields]) AND ("methylation"[MeSH Terms] OR "DNA methylation"[All Fields] OR "DNA methylations"[All Fields])
Histone modifications involved in inflammation of DN	("Inflammation"[MeSH Terms] OR "Inflammation Mediators"[MeSH Terms]) AND (("diabetes complications"[MeSH Terms] AND "kidney diseases"[MeSH Terms]) OR "Diabetic Nephropathies"[MeSH Terms] OR "Diabetic Nephropathy"[All Fields] OR "Diabetic kidney disease"[All Fields]) AND ("histones"[MeSH Terms] OR "histone"[All Fields])
Noncoding RNAs involved in inflammation of DN	("Long Noncoding "[All Fields] OR "LincRNA"[All Fields] OR "miRNA"[All Fields] OR " microRNA "[All Fields] OR "non coding RNA"[All Fields] OR "ncRNA"[All Fields]) AND ("Inflammation"[MeSH Terms] OR "Inflammation Mediators"[MeSH Terms]) AND (("diabetes complications"[MeSH Terms] AND "kidney diseases"[MeSH Terms]) OR "Diabetic Nephropathies"[MeSH Terms] OR "Diabetic kidney disease"[All Fields]))

* DN, Diabetic Nephropathy.
